# Quantitative roles of ion channel dynamics on ventricular action potential

**DOI:** 10.1080/19336950.2021.1940628

**Published:** 2021-07-16

**Authors:** Ahmet Kürşad Sırcan, Sevgi Şengül Ayan

**Affiliations:** a Department of Engineering, Electrical and Computer Engineering, Antalya Bilim University, Döşemealtı, Antalya, Turkey; b Department of Engineering, Industrial Engineering, Antalya Bilim University, Döşemealtı, Antalya, Turkey

**Keywords:** Voltage-gated ion channels, dynamics of ionic currents, contribution analysis, ventricular action potential, quantitative analysis

## Abstract

Mathematical models for the action potential (AP) generation of the electrically excitable cells including the heart are involved different mechanisms including the voltage-dependent currents with nonlinear time- and voltage-gating properties. From the shape of the AP waveforms to the duration of the refractory periods or heart rhythms are greatly affected by the functions describing the features or the quantities of these ion channels. In this work, a mathematical measure to analyze the regional contributions of voltage-gated channels is defined by dividing the AP into phases, epochs, and intervals of interest. The contribution of each time-dependent current for the newly defined cardiomyocyte model is successfully calculated and it is found that the contribution of dominant ion channels changes substantially not only for each phase but also for different regions of the cardiac AP. Besides, the defined method can also be applied in all Hodgkin–Huxley types of electrically excitable cell models to be able to understand the underlying dynamics better.

## Introduction

Cardiomyocytes are electrically excitable cells and electrical signals that are called cardiac action potentials (APs) are crucial for the excitation–contraction (E–C) of the heart [1,2]. Building steps of the E–C activity involves the building of an AP, an influx of extracellular calcium; and lastly Ca^2+^-induced Ca^2+^ release from the sarcoplasmic reticulum (SR). Cardiac APs are long APs due to the long plateau phase with a stable resting potential around −80 mV. The differences in AP waveforms and durations occur due to the differences in the underlying ionic currents. Understanding the underlying mechanisms of the spiking activity due to the various ionic currents has been an important problem experimentally and mathematically. Mathematical models of the excitable cells including the heart have been defined since the 1960s based on experimental data and the early mathematical models were based on the extensions of the Hodgkin–Huxley model that is defined for nerve conduction [3–5]. The principal mechanisms of cardiac AP analyzed with simplistic models and later realistic models are defined with more nonlinear and complex mathematical formulas [6–11].

Nowadays, computational simulations and numerical methods greatly help to mimic APs by solving such complex and nonlinear cardiac models [12]. But these complex and highly nonlinear models keep our intuitive guess in dark. Cardiac models involve several ion channels and ion transporters’ interaction together with the Ca^2+^ mechanisms and understanding the specific role of each component in this complex nonlinear network is not a straightforward task. A key challenge in the biophysical analysis of realistic models is to understand the behavior of the system, in particular, to explore the function and contribution of a particular factor out of these complex dynamics.

The specific roles of model variables for excitable systems have been explored using both qualitative and quantitative approaches. The most well-known method to determine qualitative properties of dynamic variables is bifurcation analysis also known as numerical continuation [13–16]. It basically tracks the solution by changing the parameter of interest and determines if a model converges, diverges, or oscillates. This method has been applied to several types of cardiac cell models [17–19]. While bifurcation analysis is an excellent tool for examining and describing the relationship between parameters and solutions by altering one or more parameters at a time, computational power becomes bulky as the dimension of parameter space is increased. Another good approach is sensitivity analysis [20]. Sensitivity analysis is applied to understand how sensitive the model output to changes in parameter space. Sensitivity analysis requires a broad, computationally demanding sample of the aimed parameter search space, especially when the cell model contains the higher-order nonlinear ODEs. These methods among others help us to understand how each parameter affects the change of membrane potential in excitable cell models [21,22].

Furthermore, quantitative approaches are needed to answer the question of “how much” each parameter affects the change of membrane potential. Quantitative analysis methods including dominant scale analysis [23,24], lead potential analysis [25], and relative contribution analysis [26] focus on understanding the role of each component in a complex cell model. Dominant scale analysis calculates which components are dominant in a specific time interval of AP by using an equilibrium membrane potential value. Lead potential analysis can also quantitatively calculate the contribution of each component to AP by using an equilibrium membrane potential called lead potential. But for more complex models with more ion channels will result in more complex curves of gating dynamics. Applying the contribution scale directly will not be enough to see the contributions of the activation/inactivation dynamics to AP generation. Here the contribution analysis method is defined for more complex curve dynamics and applied it to a cardiac model that has 12 ion channels defined with 32 nonlinear differential equations.

In this study, a detailed and new way of contribution measure is proposed that directly measures the contribution of each gating component to AP generation by using membrane potential (*V*) and time constants of the components (*τ*_*x*_). This novel method is employed to examine how much and where are the activation/inactivation process of ion channels contributes to the AP properties of the ventricular model of cardiac cell activity. The number of 221 model parameters are optimized according to the electrical properties of adult left ventricular cardiac cells of the rat Physical units used in the text are given in Table 1 and the resulting estimations are listed in Tables 2–3. Initial conditions and the extracellular ion concentrations are provided in Tables 4–5. The defined method successfully unveils the characteristics of each component even though the dynamics are highly nonlinear. The use of contribution analysis is also facilitated by high accuracy algorithms that are computationally efficient. It can determine the contribution of each ion channel dynamic to total membrane potential change and it can also measure the contribution of each dynamic to the specific region of AP. Moreover, the method tells us which dynamic speeds up or slows down the membrane potential in any AP phase. Since the cardiac AP waveforms vary with species, heart rate, location within the heart, developmental stage, and in response to hormones and drugs [27], the method also can be applied to quantify the channel contributions of all these different morphologies.

**Table 1.** Physical units used in the text

Time (*t*)	Voltage (*V*)	Capacitance (*Cm*)	Conductance	Concentration
Milliseconds (ms)	Millivolts(mV)	Picofarads (pF)	Nanosiemens per picofarad (nS/pF)	Millimol per liter (mmol/liter)

**Table 2.** Conductances of ion channels

Parameter	Value	Parameter	Value
$g_{Na}$	1.0044e+3	$g_{Ks}$	6.4916
$g_{t}$	16.4718	$g_{CaT}$	1.5854
$g_{hf}$	7.7282	$g_{Clb}$	1.5278
$g_{K1}$	6	$g_{BNa}$	0.0326
$g_{Kr}$	5.7017	$g_{BK}$	0.0500
$g_{CaL}$	6.2894	$g_{BCa}$	0.0025

**Table 3.** Membrane current and Ca^2+^ handling mechanism related parameters for equations above

Parameter	Value	Parameter	Value	Parameter	Value
$k_{b}^{-}$ (ms^−1^)	3.4472	$v_{maxr}$(mM ms^−1^)	0.0009	$k_{htrpn}^{+}$(mM^−1^.ms^−1^)	43.1286
$k_{c}^{+}$ (ms^−1^)	0.0513	$K_{m}^{CMDN}$ (mM)	0.00238	$k_{htrpn}^{-}$(ms^−1^)	7.1033e-4
$k_{c}^{-}$ (ms^−1^)	0.0062	$K_{m}^{CSQN}$ (mM)	0.8	$k_{ltrpn}^{+}$(ms^−1^)	0.0969
$k_{a}^{+}$(mM^−4^ ms^−1^)	5.78e+6	${\left[CMDN\right]}_{tot}$(mM)	0.05	$k_{ltrpn}^{-}$(ms^−1^)	0.0013
$k_{a}^{-}$ (ms^−1^)	0.5128	${\left[CSQN\right]}_{tot}$(mM)	15	${\left[HTRPN\right]}_{tot}$(mM)	0.14
$k_{b}^{+}$(mM^−3^ ms^−1^)	2.1586e+7	$k_{EGTA}^{-}$ (mM)	0.00015	${\left[LTRPN\right]}_{tot}$(mM)	0.07
v_1_ (ms^−1^)	10.5921	$EGTA_{tot}$(mM)	10	$V_{myo}$(pL)	9.36
$K_{fb}$ (mM)	9.6176e-4	$V_{JSR}$(pL)	0.056	*F* (Faraday Constant-C/mol)	96,487
$K_{rb}$ (mM)	28.1467	$V_{ss}$(pL)	0.0012	*T* (Absolute temperature – K)	310.15
$K_{sr}$	2.3974	$V_{NSR}$(pL)	0.504	*R* (Ideal gas constant –mJ/mol K)	8314
$N_{fb}$	1.2	$A_{cap}$	0.8004		
$N_{rb}$	1	$\tau_{tr}$ (ms)	0.9258		
$v_{maxf}$(mM ms^−1^)	0.00004	$\tau_{xfer}$(ms)	0.5234

**Table 4.** Initial conditions for state variables

Parameter	Initial value	Parameter	Initial value
V	−72	$P_{C_{2}}$	0.634
n	0.0041	HTRPNCa	0.139
$n_{f}$	0.67	LTRPNCa	0.00516
$n_{s}$	0.67	${\left[Na^{+}\right]}_{i}$	10.73519
l	2.1 × 10^−6^	${\left[K^{+}\right]}_{i}$	139.275
$l_{f}$	0.99	${\left[Ca^{2}+\right]}_{i}$	7.9 × 10^−5^
$l_{s}$	0.99	${\left[Ca^{2}+\right]}_{ss}$	8.737212 × 10^−5^
$Ca_{i}$	0.99	${\left[Ca^{2}+\right]}_{JSR}$	6.607948 × 10^−2^
kt	0.0021	${\left[Ca^{2}+\right]}_{NSR}$	0.06600742
$kt_{f}$	0.98	r	0.3657
$kt_{s}$	0.64	ct	0.089
y	0.0035	$ct_{i}$	0.081
$P_{C_{1}}$	0.634	$Cl_{i}$	30.03
$P_{O_{1}}$	4.327 × 10^−4^	s	0.1776
$P_{O_{2}}$	6.06 × 10^−10^	$r_{i}$	0.1

**Table 5.** Extracellular ion concentrations

Parameter	${\left[Na^{+}\right]}_{o}$	${\left[K^{+}\right]}_{o}$	${\left[Ca^{2}+\right]}_{o}$	${\left[Cl^{-}\right]}_{o}$
Value (mM)	140	5.4	1.5	140

## Methods

### Model description

The applied model is based on a recently defined cardiomyocyte model [11]. It involves time-dependent and independent ionic currents, and the related parameters were optimized by voltage-clamp experiments using adult rat ventricular cells. The model consists of 32 nonlinear differential equations governing the dynamics of 8 ionic currents, 4 ionic background currents, 3 pump current, and a Ca^2+^ handling mechanism.

The membrane voltage *V* is expressed in terms of the sum of all currents passing through the membrane:

(1) $$C_{m}\frac{dV}{dt}=-I_{Na}-I_{CaL}-I_{CaT}-I_{t0}-I_{K1}-I_{Kr}-I_{Ks}-I_{f}-I_{BCa}-I_{BNa}-I_{BK}-I_{BCl}-I_{NaK}-I_{NaCa}-I_{CaP}+I_{app}$$

for a membrane capacitance $C_{m}$ and an applied current $I_{app}$.

All ionic currents in the model fit the following formula;

(2) $$I_{Y}=\bar{g_{Y}}a^{M}b^{N}c^{L}\left(V-E_{ion}\right),Y=Na,CaL,CaT,t_{0},K_{1},K_{r},K_{s},f,K_{b},Ca_{b},Na_{b},Cl_{b}$$

Here *Y* is corresponding ion channel, $\bar{g_{Y}}$ is channel maximal conductance for the reversal potential $E_{ion}$ (ion = Na^+^,Ca^2+^,K^+^,Cl^−^). *a,b,c* represents activation, fast inactivation, and if present, slow inactivation gating variables, respectively. *M*, *N*, *L* are the corresponding integer powers of those gating variables. Each voltage-dependent gating variable is given by

(3) $$\frac{dx}{dt}=\frac{\left(x_{\infty }\left(V\right)-x\right)}{\tau_{x}\left(V\right)},x=n,n_{f},n_{s},l,l_{f},l_{s},Ca_{i},c_{t},c_{ti},k_{t},k_{tf},k_{ts},r,r_{i},s,y.$$

In Equation 3, once the channel activation/inactivation is so fast meant $\tau_{x}$ is so small, gates approach the equilibrium ($x≅x_{\infty }$). So, 1/τ represents a time-dependent “eigenvalue” approaching a time-dependent asymptotic target. Each steady-state function is denoted by:

(4) $$x_{\infty }\left(V\right)=\frac{1}{1+e^{a_{1}V+a_{2}}},x=n,n_{f},n_{s},l,l_{f},l_{s},Ca_{i},c_{t},c_{ti},k_{t},k_{tf},k_{ts},r,r_{i},s,y.$$

And each time constant is given as:

(5) $$\tau_{x}\left(V\right)=\frac{1}{\sum_{i=1}^{n}a_{3i}e^{a_{3i+1}V+a_{3i+2}}},i=1,2,\ldots ,n.$$

In the defined method here, time constant $\tau_{x}$ is perturbed and the related change in the output on the AP curve is calculated as a measure to “contribution” of the related ion channel.

The contributions of each ion channel are analyzed in this manner for the time-dependent currents in the model listed as (1) *I*_*Na*_ is the fast Na^+^ current with activation *n*, fast inactivation *n_f_* and slow inactivation *n_s_*; (2) *I*_*CaL*_ is the L-type Ca^2+^ current with activation *l*, fast inactivation *l_f_*, slow inactivation *l_s_* and Ca^2+^ dependent inactivation *l_Cai_*; (3) *I*_*CaT*_ is the T-type Ca^2+^ current with activation *ct* and inactivation *cti*; (4) *I*_*to*_ is the transient K^+^ current with activation *kt*, fast inactivation *kt_f_* and slow inactivation kt_s_; (5) *I*_*Kr*_ is the rapidly activating delayed rectifier K^+^ current with activation *r*, inactivation *r_i_*; (6) *I*_*Ks*_ is the slowly activating delayed rectifier K^+^ current with activation *s*, and (7) *I*_*f*_ is the hyperpolarization-activated current with activation variable *y*. Full mathematical model details are given in Appendix.

### Description of the novel method

**Figure 1.**
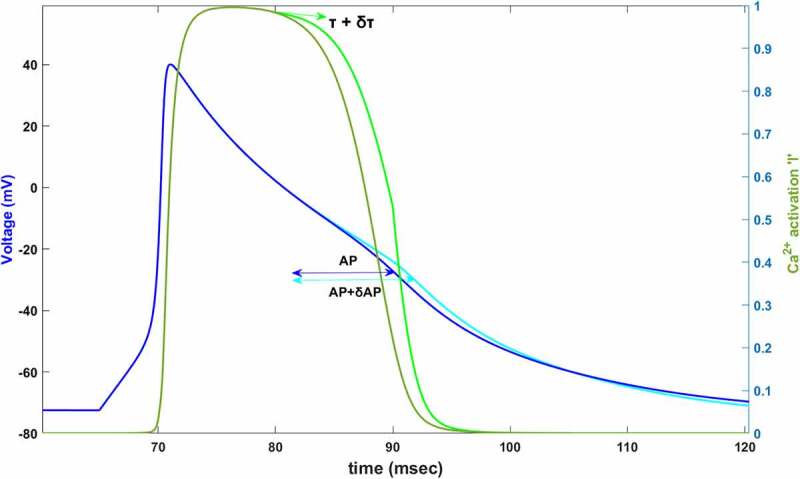
Simulated AP (dark blue) and simulated Ca^2+^ activation function,l, (dark green) between 60–120 ms. In 80 mV, time constant of Ca^2+^ activation $\tau_{l}$ is increased by *δτ* (light green). Slowing down the Ca^2+^ activation (%60 for this plot) increases AP duration by δAP (light blue)

Complex interactions between ion channels are resulted in producing the cardiac AP in the so-far-defined models [8,9,11,27]. We wish to know the relative contributions of each voltage-gated state variable to AP termination to be able to understand the dynamics under the complex interactions. The main idea here is changing the speed of the voltage-gated state variable by changing its time constant will affect the AP duration. As shown in Figure 1, we find the relative contribution of each channel’s activation/inactivation, let’s say *x*, to cardiac AP by perturbing its time constant $\tau_{x}$ and calculating the fractional change in the duration of AP as ∂AP. In order to measure the contribution of *x* to the AP, in general, time constant *τ_x_* is perturbed at the beginning of a specific time interval then the percentage of change that occurs is calculated in the AP duration according to the equation:

(6) $$C_{AP}^{x}=\frac{\partial AP}{AP}\frac{\tau_{x}}{\partial \tau_{x}}.$$

These complex and nonlinear dynamics are firstly analyzed by defining the “epochs” by considering both the geometry of the AP and activation/inactivation curves. Later AP can be divided into phases and phases can be divided into intervals of interests (IoI) to reach the desired level of details. Moreover, the sign convention of the method is defined here. To apply it, the time constant *τ_x_* is perturbed as 10% (*δτ_x_* = 0.1 *τ_x_*) at the beginning of each epoch point. Note that, 10% value is chosen large enough to be able to see the change in the AP duration. Our method can successfully find the contribution of each state variable modeled as in Equation 3. Because of the desired level of detail in our analysis, all defined regions are explained in detail below.

#### AP Phases

The ventricular AP results from the flow of ions through ion channels and the associated waveform is generally defined with five phases from phase 0 to phase 4. Phase 0 is the depolarization phase and the observed rapid upstroke is primarily due to the Na^+^ channel activation (*n*). Phase 1 is known as the early rapid repolarization resulting in Na^+^ inactivation (*nf, ns*) together with K^+^ activation (*kt*). Phase 2 is the plateau phase and K^+^ channels are balanced with Ca^2+^ channels to create this plateau in cardiac AP. The final rapid depolarization is happening at phase 3 with the inactivation of the Ca^2+^ channels. And lastly, diastolic depolarization is known as phase 4 where the Na^+^ current balance the K^+^ currents, but we will not focus on this phase separately instead we will combine it with phase 3.

**Figure 2.**
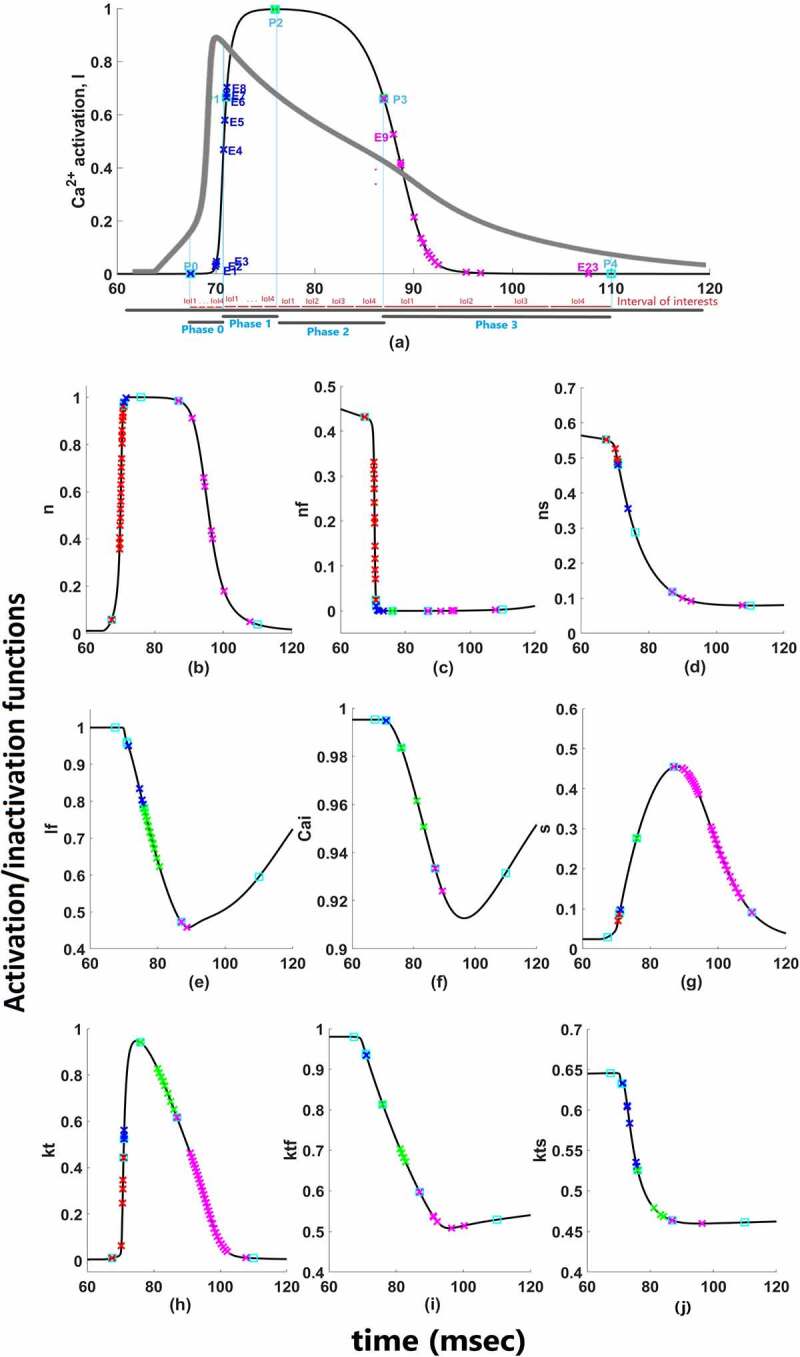
Functions for time-dependent (a) activation of *I*_*Ca*_*_L_;* (b) activation of *I*_*Na*_; (c) fast-inactivation of *I_Na_*; (d) slow-inactivation of *I*_*Na*_; (e) fast-inactivation of I_CaL_; (f) Ca^2+^ depedendent-inactivation of *I*_*CaL*_; (g) activation of *I*_*Ks*_; (h) activation of *I*_*to*_; (i) fast-inactivation of *I*_*to*_; (j) slow-inactivation of *I*_*to*_. Red marks show epoch points of phase 0, blue marks show epoch points of phase 1, green marks show epoch points of phase 2 and pink marks show epoch points of phase 3. Light blue squares are phase points on each curve

There are two different Ca^2+^ channels in our model as T-type and L-type Ca^2+^ channels and 4 types of K^+^ channels as *I*_*to*_, *I_Kr_*, *I*_*Ks*_, and *I*_*K1*_. Moreover, these currents have several activation and inactivation gates. Complex nonlinear interaction hinders our intuitive understanding of their quantitative roles for cardiac AP. So which channel’s activation or inactivation contributes to which phase and how much are the question we would like to answer at the end of our contribution analysis. To answer these questions, first, the simulated cardiac AP is divided into four phases as shown in Figure 2a. Cardiac depolarization is phase 0 and shown from point P0 to P1, early repolarization is phase 1 and shown from point P1 to P2, plateau phase is phase 2 and shown from P2 to P3 and lastly, late repolarization is phase 3 and shown from point P3 to P4. As long as the curve of AP and the focused activation/inactivation curves are increasing or decreasing linearly between the phase points, the contribution can be measured with one perturbation applied at the beginning of the phase with Equation 6. But as shown in Figure 2a, between the phase points both AP curve and activation/inactivation curves of ion channels have several inflection points means that the applied perturbation will not affect the duration linearly. That is why we need to derive the *Epochs* by dividing the phases from the inflection points where the curves have “zero” as a second derivative. Phases are user dependent and one can choose to analyze according to the AP duration on different percentages too, but epochs are the main mean of the contribution quantification.

#### Epochs

Epochs are the intervals between two inflection points. The time-varying epoch points are calculated along a numerically computed trajectory of cardiac AP as $\frac{d^{2}V}{dt^{2}}=0$ and $\frac{d^{2}x}{dt^{2}}=0$. Let’s say, in order to find the contribution of gating variable *x* to phase 0 which we show as $\;C_{AP0}^{x}$, the time constant of *x*, $\tau_{x},$ is perturbed at the beginning of phase 0 (P0) and the fractional change in AP duration is calculated at the end of phase 0 (P1) as long as the epochs are not derived between the phase points. Epochs are derived either when (a) the second derivative of gating curves is zero or (b) the second derivative of the AP curve is zero. If the second derivative of *V* or *x* changes sign means if *x* has some inflection points, the phase is divided into *epochs* from these inflection points. Epoch can be defined as the interval between two inflection points as shown in Figure 2a with E1, E2 and, E3. Then, the contribution of *x* can be calculated for each epoch by perturbing the time constant at the beginning of each epoch and calculating the AP elongation until the end of the epoch. Then the total contribution of *x* to phase 0 is calculated with aggregated n epochs. For Ca^2+^ activation as an example given in Figure 2a, the contribution is given as follows:

(7) $$C_{AP0}^{l}\;=\;\overset{3}{\underset{k=1}{\sum }}\frac{\partial E_{k}}{E_{k}}\frac{\tau_{l}}{\partial \tau_{l}}$$

Similarly, for other phases as shown in Figure 2b with P0, …, P5 total contribution of *l* to phase 1,2 and 3 is measured by perturbing $\tau_{x}$at the beginning of each epoch (inflection points) is given by;

(8) $$C_{AP1}^{l}\;=\;\overset{8}{\underset{k=4}{\sum }}\frac{\partial AP1_{k}}{AP1_{k}}\frac{\tau_{l}}{\partial \tau_{l}}$$

(9) $$C_{AP2}^{l}\;=\;\overset{12}{\underset{k=9}{\sum }}\frac{\partial E_{k}}{E_{k}}\frac{\tau_{l}}{\partial \tau_{l}}$$

(10) $$C_{AP3}^{l}\;=\;\overset{27}{\underset{k=13}{\sum }}\frac{\partial E_{k}}{E_{k}}\frac{\tau_{l}}{\partial \tau_{l}}$$

∂ shows change in the duration of the time in equations. Phase contribution quantification depends on the epochs as shown in Equations 7, 8, 9 and 10. The numerical calculations for determining epochs are detailed in the Simulations part.

#### Interval of interests

For cardiac models, we find the contributions of each phase by dividing the phase into epochs from the inflection points. Besides that, we can find the contribution of each state variable to a specific time interval of AP which we can choose. To find the contribution of a state variable to our interval of interest (IoI), phases can also be divided into portions. We can find the relative contribution for each portion by using Equation 11 which is similar to Equation 6. For example, to be able to see the detailed contributions during the peak overshoot, or plateau, we can divide phase 2 into the interval of interests. To find the total contribution for more than one portion, the fractional change in total portion durations is calculated by Equation 12.

(11) $$C_{i}^{x}\;=\;\frac{\partial P}{P}\frac{\tau_{x}}{\partial \tau_{x}}$$

(12) $$C_{Total}^{x}\;=\;\frac{\sum_{k=1}^{n}\partial P_{k}}{\sum_{k=1}^{n}P_{k}}\frac{\tau_{x}}{\partial \tau_{x}}$$

Moreover, there can be many epochs over the course of a typical AP trajectory and simulating very close epochs may be relatively unimportant or may result in big numerical errors. So we can aggregate similar or short-lived epochs into a few IoIs. To calculate the focused IoIs, we should sum the related epoch points. This means that aggregation possibilities are generally not unique, and thus IoIs’ derivation is model and user dependent.

Here, phases of AP are divided into four equal portions that form our IoIs. As shown in Figure 2a, the calculations are done based on the epochs lying under each IoI in our simulations.

#### Sign convention of the method

Here, the *C*_*x*_ values are computed as the contribution values of each channels’ activation/inactivation processes based on Equation 6. Once we apply the contribution measure, negative or positive contribution values can be observed depending on the effect of the corresponding gating variable. During the AP duration, increasing and decreasing behaviors appear for both activation and inactivation curves as shown in Figure 2.

**Figure 3.**
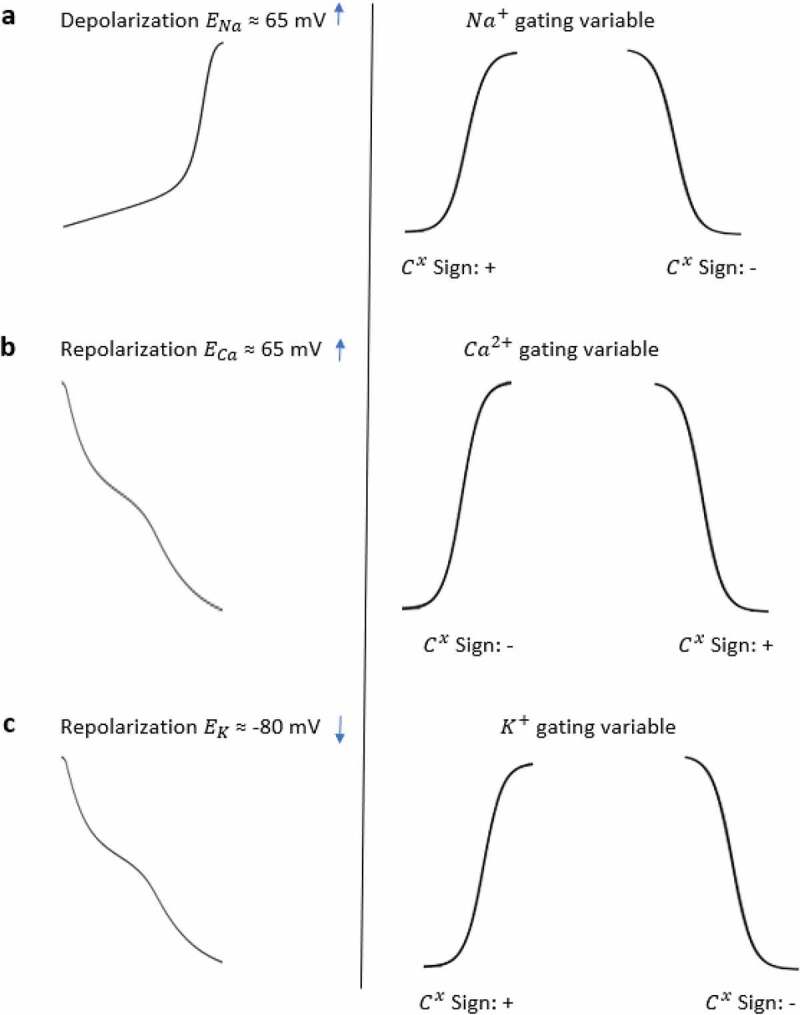
Sign convention of the contribution analysis method. The traces of (a) show sign of contribution value of Na^+^ gating variables under the effect of Na^+^ Nernst potential while membrane is in depolarization state. The traces of (b) show sign of contribution value of Ca^2+^ gating variables under the effect of *Ca*^2+^ Nernst potential in repolarization state and the traces of (c) show sign of contribution value of K^+^ gating variables under the effect of K^+^ Nernst potential in repolarization state

Regardless of the activation and inactivation gate, if the AP curve is in the same direction as the gating curve and the Nernst potential of the channel is positive, we find a positive contribution value at the end of the analysis. Examples of positive contributions with the channels providing positive feedback to the system are given in Figure 3a with increased Na^+^ transition during depolarization and in Figure 3b with reduced Ca^2+^ transition during repolarization. However, if the AP curve is in the opposite direction with the gating curve and the Nernst potential of the channel is positive, a negative contribution value is reached. Figure 3a shows such an example with decreased Na^+^ transition during depolarization as well as Figure 3b shows increased Ca^2+^ transition during repolarization. Since K^+^ has an opposite effect with negative Nernst potential, we can see the signs will be vice versa in Figure 3c.

The sign of contribution values basically tells us if a gating variable speeds up or slows down the AP in the corresponding epoch. Since activation and inactivation curves are not always opposite to each other in every epoch, that is why we cannot say that the activation and inactivation gating variables’ contribution always has opposite signs. Figure 3 summarizes all the sign conventions of the interested channel gating for the focused model. Since we aimed for a detailed analysis here we consider both fast and slow inactivation gating variables together with associated activation variables separately for each ion channel.

### Simulations

The model consists of 32 nonlinear differential equations. The stability ranges of these differential equations are very small because of the high difference between coefficients in the model therefore the system is highly stiff. One of the most common and efficient stiff differential equation solving methods, the Gear Method [28], was used to solve model differential equation system. The Gear method is an efficient method to solve stiff systems because it adjusts step size to its best value in every iteration. Ventricular AP in our model is generated with an applied current of 528 pA for 5 seconds which is adapted with the experimental protocol used to evoke APs in rat ventricular myocytes. After solving the differential equation system with the Gear method, we perform contribution analysis simulations with a step size fixed to 0.0001. The step size is chosen small enough to see even the contribution of very small currents like *I*_*f*_, *I_CaT_,* and *I*_*Kr*_. Moreover, it is crucial to take small steps to catch all the inflection points even on the flat regions of the AP curve like the plateau phase. All calculations were performed in a MATLAB environment. All time-dependent gating variables are explicitly simulated and the results of inflection points, related epochs, and defined IoIs are shown in Figure 2.

## Results

**Figure 4.**
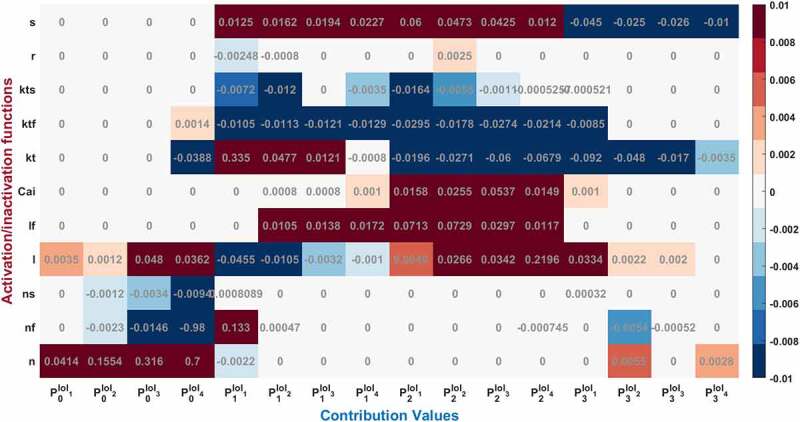
Contribution results after the simulations are defined as a heatmap between −0.01 and 0.01. Each phase (P_0,1,2,3_) is divided into four equal interval of interests (IoI_1,2,3,4_) calculated as aggregated epochs. Each time dependent function in the model that has a role to shape cardiac action potential is measured and their roles are quantified in the table

**Figure 5.**
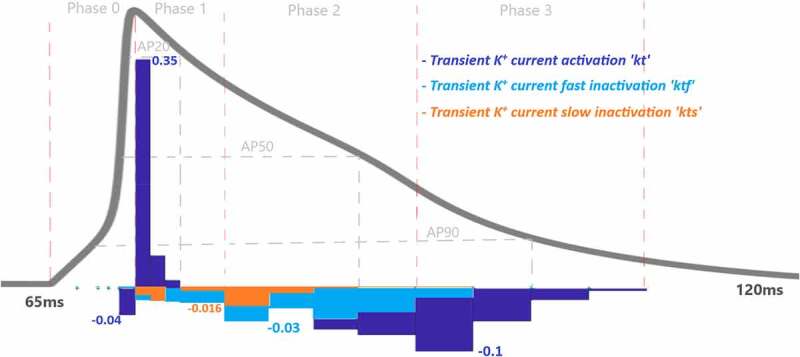
Contribution quantification for Na^+^ current activation “n”, fast inactivation “nf” and slow inactivation ‘ns’during ventricular AP depolarization (phase 0 is between 67.44 and70.92 ms), early repolarization (phase 1 is between 70.92 and76 ms), plateau (phase 2 is between 76 and 90.76 ms) and late repolarization (phase 3 is between 90.76 and110 ms). Regions for the action potential duration of %20 (AP20), %50 (AP50) and %90 (AP90) are also shown. All phases are divided into four equal IoI regions as color coded

**Figure 6.**
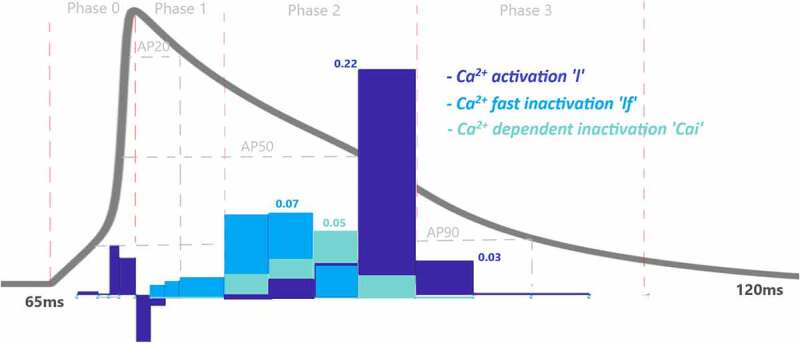
Contribution quantification for Ca^2+^ current activation “l”, fast inactivation “lf” and Ca^2+^ dependent inactivation “Cai” during ventricular AP depolarization (phase 0), early repolarization (phase 1), plateau (phase 2) and late repolarization (phase 3). Regions for the action potential duration of %20 (AP20), %50 (AP50) and %90 (AP90) are also shown. All phases are divided into four IoI regions as color coded

**Figure 7.**
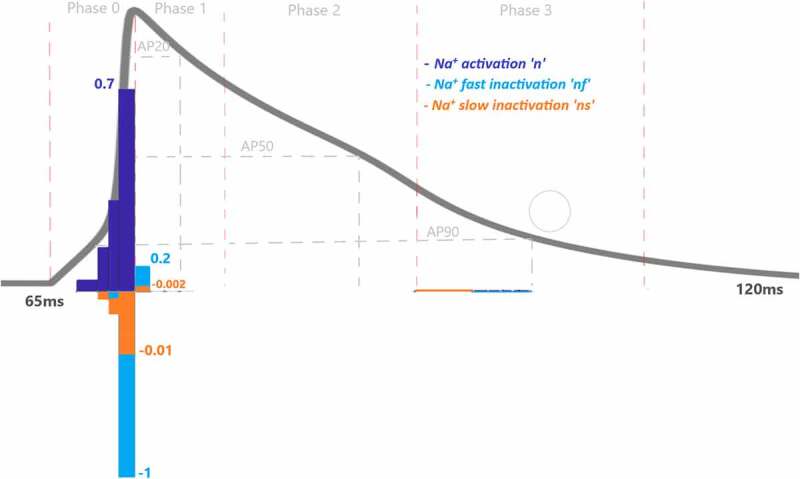
Contribution quantification for K^+^ current activation “kt”, fast inactivation “ktf” and slow inactivation “kts” during ventricular AP depolarization (phase 0), early repolarization (phase 1), plateau (phase 2) and late repolarization (phase 3). Regions for the action potential duration of %20 (AP20), %50 (AP50) and %90 (AP90) are also shown. All phases are divided into four IoI regions as color coded

**Figure 8.**
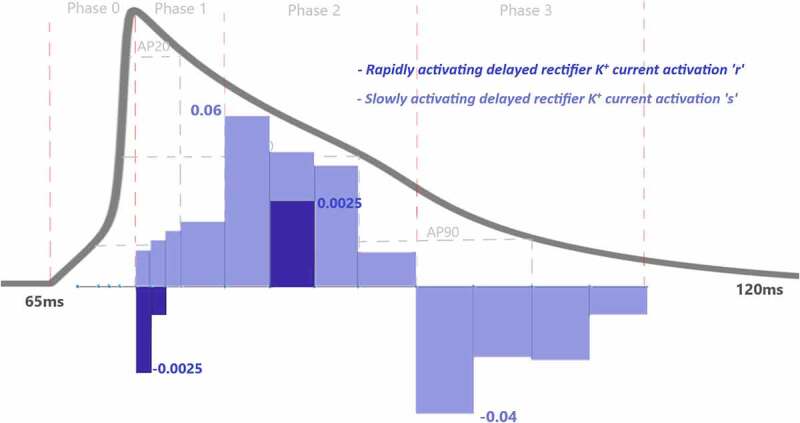
Contribution quantification for rapidly activating delayed rectifier K^+^ current activation “r” and slowly activating delayed rectifier K^+^ current activation “s” during ventricular AP depolarization (phase 0), early repolarization (phase 1), plateau (phase 2) and late repolarization (phase 3). Regions for the action potential duration of %20 (AP20), %50 (AP50) and %90 (AP90) are also shown. All phases are divided into four IoI regions as color coded

To assess the contributions of each activation and inactivation of ion channels for the ventricular AP, first epochs are calculated from the inflection points of the curves of AP and gating functions as shown in Figure 2. Epochs are the basic points of the calculation for our analysis. Later, the regions that will be focused are user dependent. Since we would like to analyze the roles of each gating function during the phases of the AP, phases are determined according to the depolarization (P0), the early repolarization (P1), the plateau phase (P2), and the late repolarization (P3). Moreover, each phase is also divided into four equal parts as intervals of interest to be able to observe the changes of the roles during the course of each phase. Figure 4 is a heatmap of the contribution results colored for between −0.01 and 0.01. Besides that, AP duration (APD) at 20%, 50%, and 90% of the total AP duration are measured and analyzed in many animal/human models to better understand the AP behavior. So, the roles of the ion channels are compared during APD at 20, 50, and 90% repolarization in Figures 5–8. All contributions are calculated as the aggregated sum of the related epochs lying under the focused region. The contribution results of the epochs are combined for each current under the related IoIs, phases, and APDs as shown in Figures 5–8. Each phase is also analyzed separately in Supplementary Figures 1–4.

While the dominant role of Na^+^ activation *n* can be seen during phase 0 in Figure 4, the increasing contribution can also be observed until the end of the phase by checking the values from $P_{0}^{IoI_{1}}$ to $P_{0}^{IoI_{4}}$ [0.0414, 0.1554, 0.316, 0.7]. Fast inactivation of Na^+^ channel has the highest activity at the end of the Phase 0 with $C_{P_{0}^{IoI_{4}}}^{nf}=-0.98$ and later it is nearly zero. Gating functions of the K^+^ channels seem so busy during P1 and P2 and Ca^2+^ has the biggest impact over P2. To better understand and explain the underlying behavior of the nonlinear cardiac model, we identified the contributed channels for each phase and explain them below.

### Phase 0: Cardiac AP depolarization

The contribution analysis method successfully measures the total contribution of each dynamic to cardiac AP depolarization. Moreover, the method can calculate the contribution of each dynamic to any specific region of AP during the depolarization. The results in Figures 5–8 reveal the characteristics of all time-dependent gating variables in cardiac AP phases namely determination of phases, the effect of Nernst Potentials and how depolarization and repolarization start and end in the cell membrane.

Figure 5 shows $C_{AP}^{x}$ values for the Na^+^ current driving the AP. Depolarization is mostly determined by Na^+^ channel activation with the largest $C_{AP}^{n}=0.96$ value as expected. There are three gating variables that control the ionic movements of I_Na_ in the model (activation n, fast inactivation nf, slow inactivation ns). The method measures the total contribution of Na^+^ activation (n) as $C_{AP}^{n}=0.96$, Na^+^ fast inactivation (nf) as $C_{AP}^{nf}=-0.99$ and Na^+^ slow inactivation (ns) as $C_{AP}^{ns}=-0.014\;$to depolarization phase. Phases are also divided by four IoIs to better analyze where this activation is more effective in this phase. We should point out that these IoIs capture different epochs and all $C_{AP}^{X}$ results are the cumulative sum of the included epochs. IoI bar graphs show us the role of Na^+^ activation is increasing until the end of phase 0. The role of Na^+^ inactivation is also gradually increasing until the end of the phase 0 as shown in $C_{AP}^{nf}$ and $C_{AP}^{ns}$ bar graphs.

Moreover, the positive sign of Na^+^ activation *n* shows that it is responsible to open Na^+^ channel which provides positive feedback to the system in phase 0 and tries to depolarize the membrane current. On the other hand, the negative signs of inactivation gates, *ns* and *nf*, show they are closing the Na^+^ ion gates and provides negative feedback to the depolarization phase, so that the membrane ends depolarizing and starts to repolarize the AP.

The L-type Ca^2+^ activation gating variable (*l*) also contribute to phase 0 as $C_{AP}^{l}$ = 0.11 as seen in Figure 6 and SF1. There is also a very small almost negligible role of transient current (*I*_*to*_) activation (*kt*) and fast inactivation (*ktf*) to phase 0 with $C_{AP}^{kt}$ = −0.03 and$C_{AP}^{ktf}=0.003$, respectively as shown in Figure 7.

These results show that Na^+^ activation fires AP to −15 mV with the help of small contribution of L-type Ca^2+^ activation. Activation of the channels’ contributions increase mostly to the second part of the depolarization phase in which membrane voltage fires up to the pick value 40 mV (transition to repolarization from depolarization). Similarly, the positive sign of *l* provides positive feedback by activating the L-type Ca^2+^ channels and the negative sign of transient K^+^ activation, *kt*, provides negative feedback to this nonlinear system in phase 0.

### Phase 1: Cardiac AP Early Repolarization

As a result of contribution analysis, the beginning of the repolarization phase mostly driven by the transient K^+^ current activation (*kt*) with $C_{AP}^{kt}$ = 0.34 and then decreased until the end of phase 1 to $C_{AP}^{kt}$ ≈ 0 as seen in Figure 7.

Na^+^ inactivation gate is also still active at the very beginning of this phase (Figure 5, SF2) and has a role to decrease the AP from 40 mV to 39 mV with the value of $C_{AP}^{nf}$= 0.13. There are also small contributions of L-type activation and fast inactivation gating variable to this phase as $C_{AP}^{l}$= −0.06 and $C_{AP}^{lf}$= 0.03 as shown in Figure 6 and SF2.

Here the negative $C_{AP}^{x}$ sign occurs in the activation gate of L-type Ca^2+^ channel and positive sign occurs in the inactivation gate. Because the Nernst potential of Ca^2+^ activation tries to depolarize the membrane while the membrane is repolarizing so that it provides negative feedback to the system. The inactivation gate of L-type Ca^2+^, on the other hand, works in the opposite way providing positive feedback to the repolarization.

Figure 8 shows that slowly activated K^+^ outward current (*ks*) activation has also a role to shape the repolarization and the contribution of $C_{AP}^{s}$ is increasing this time in the course of the phase. Another K^+^ current in the model is rapidly activated outward current and the contribution of I_Kr_ activation is almost 20 times smaller than I_KS_ to phase 1 at the beginning of the repolarization.

### Phase 2: Cardiac AP Plateau Phase

Phase 2 of the cardiac AP is known as the plateau phase which depends on the delicate harmony of Ca^2+^ and Na^+^ influx and K^+^ efflux of the cell. As shown in Figures 5–8 and SF 1–4 various ion channels contributing to phase 2. In Figure 6, Ca^2+^ influx through high-threshold, L-type voltage-gated Ca^2+^ channels is the main contributor of inward current during the plateau phase with $C_{AP}^{l}=0.22.$ Also, I_CaL_ undergoes Ca^2+^-dependent inactivation (CDI) and voltage-dependent inactivation (VDI) with the values of $C_{AP}^{Cai}=0.014$and$C_{AP}^{lf}=$0.011, respectively. As previous experimental works suggested for humans [29,30] and newborn rat [31], CDI is the major mechanism controlling the Ca^2+^ current and maintaining the plateau potential [32]. Our analysis revealed that at the end of phase 2 CDI has a dominant role in the model that is in good agreement with the results, but at the beginning of the phase VDI contribution is higher. This opposing result can be due to the model that does not capture the Ca^2+^ mechanism well, and it should be calibrating as a result of the channel contributions. Since the model is defined for adult ventricular cells, the roles of the CDI/VDI balance can also be specie-dependent. More simulation results in the model on CDI/VDI balance are included in the discussion.

As the Ca^2+^ channels inactivate, the outward K^+^ currents predominate as we see with the contribution bars for I_to_ activation with a value of$C_{AP}^{kt}=-0.12$ in Figure 7 and for slow and rapid activated K^+^ currents activations with the values of $C_{AP}^{s}=0.17$ and $C_{AP}^{r}=0.002$ in Figure 8. Just as the activation of K^+^ efflux role during the plateau, inactivation of transient and rapid activated K^+^ channels are also active in this phase with contributions $C_{AP}^{ri}=-0.002$ and $C_{AP}^{ktf}=-0.06$ and $C_{AP}^{kts}=-0.02$. Additionally, late sodium current starts flowing after the peak contributes to maintaining and prolonging the AP plateau and we can start to see its role here with $C_{AP}^{nf}=-0.0007$ shown in Figure 5. Even though the contribution looks small for this late (slowly inactivating) Na^+^ current, several works stated its importance in terms of the novel anti‐arrhythmic agents [33–38].

### Phase 3: Cardiac AP Late Repolarization

Phase 3 is known as late repolarization that is mostly driven by the activation of the time‐ and voltage‐dependent K^+^ currents. As seen in Figures 7–8 and SF 4, transient and slowly activated K^+^ channel activations are mainly responsible for this phase with $C_{AP}^{kt}=-0.11$ and$C_{AP}^{s}=-0.12$. The negative sign of $C_{AP}^{s}$ value tells us that it provides negative feedback and helps AP to reach a resting state. Note that, slowly-activated K^+^ channels do not have an inactivation gate in the model. The surprising result of this phase is the contribution value of the Ca^2+^ activation gating variable (*l*) which is $C_{AP}^{l}=$0.03 in Figure 6. Phase 3 is mainly known as the phase in which K^+^ channels closes and Ca^2+^ and Na^+^ channels start to depolarize membrane toward resting potential. The positive contribution value of L-type Ca^2+^ activation means it behaves like an inactivation gating variable. The nature of gating variables can let this situation happen. Note that, its contribution is mostly to between −25 mV and −35 mV where the plateau phase ends and phase 3 starts. This is a consistent result with the biophysical characteristics of these channels. The contribution analysis can show even the small contributions like Na^+^ channel with $C_{AP}^{nf}=-0.007$and $C_{AP}^{ns}=0.007$ in this phase. It is important to recognize that both early and late repolarization are highly non‐linear, and for different pathological conditions different channel contributions can be observed, and quantitative analysis is a great help to solve the puzzle under this complexity. We also observed a slight role of T-type Ca^2+^ channels in this phase with a contribution $C_{AP}^{ct}=-0.001$. The currents that are not shown in Figures 5, 6, 7, and 8 have either negligible or no contributions.

## Discussion

APs are electrical signals that carry information around our body. Some AP waveforms have short durations as in neurons and some have longer AP durations as in the ventricular APs. Plateau potentials that form the phase 2 and resting membrane potentials that form phase 4 can also vary as higher or lower potentials. These differences in waveforms matter for the functioning of the excitable cells. For example, the slow repolarization is crucial for appropriate excitation–contraction coupling in cardiac muscle, and electrical stability is controlled by explicit regulation of the AP duration. AP phases of the ventricular AP are driven by complex interactions of the time- and voltage-dependent properties of the underlying voltage-gated currents. Thus, alterations in the characteristics or the amounts of these channels could end with extreme effects on ventricular AP phases. That is why understanding the mechanism underlying the cardiac AP phases precisely helps us to understand the physiological and pathological conditions.

In this study, we defined a new method to determine the quantitative contribution of each voltage-gated state variable to cardiac AP generation. Our method relies on the effect of an applied perturbation to the time variable on the AP duration. To get a precise factor of duration changes, we need to make sure that both the AP curve and the interested gating curve should increase or decrease linearly. According to the AP shape, our method divides the AP waveform from the inflection points and forms the epochs. Inflection points of gating curves forms what we called the epochs. Phases and epochs are model-dependent regions in our method. Moreover, we can define interval of interests for particular region of AP as user-dependent analysis for the models. After applying enough perturbation to time constants at the beginning of each defined region including phase points and epoch points, the increase or decrease in the duration of the region is calculated. IoI is later calculated as the aggregated sum of the related epochs lying under the IoI. The sign convention of the method is also defined according to the gating variable whether it helps to increase the duration of the defined region or the opposite. Our method is applied to analyze the adult rat ventricular cardiac membrane excitation and the results could provide valuable intuition concerning the explanation of the nonlinear interactions between the dynamics of the distinct ion channels.

There are various K^+^ currents and two different types of Ca^2+^ currents in ventricular myocytes with different activation/inactivation gates. Our method confirms that the rapid depolarization or phase 0 of the cardiac AP is driven by Na^+^ current (*I*_*Na*_) (*n,nf,ns*), and causes voltage-dependent activation (*l*) of *I*_*CaL*_. Also, the dominant roles of *I*_*to*_ activation during the early repolarization, *I*_*CaL*_ activation during the plateau phase and again *I*_*to*_ activation for the late repolarization phase are quantified as a result of the analysis. In addition, we revealed the roles of the other Ca^2+^ and K^+^ channels during the phases such as *I*_*Ks*_ activation has also a role to shape the repolarization and its contribution is increasing during phase 1. Also, the balance of the IK and ICa channels during the plateau phase together with the contribution of the late Na+ current during the late repolarization is quantified.

Once the contribution analysis is applied to the rat ventricular model for the first time, the results were able to catch the part of the Ca^2+^ process but the role of the VDI was larger than the CDI at all parts of phase 3 that was opposed to works of [30–33]. The method agreed that the intracellular [Ca^2+^] ([Ca^2+^]_i_) begin the processes of VDI and CDI at phase 0, and they are dominant during phase 2 causing a progressive decrease in *I*_*CaL*_. But to regulate the roles of the VDI–CDI ratio, [Ca^2+^]_i_ and calmodulin level is increased in the model so that the dominant role of CDI could be observed. Moreover, the Ca^2+^ transient duration is shorter than the physiological AP duration in the model [39]. So the analysis results reveal that the Ca^2+^ mechanism should be calibrated and the Ca^2+^ process should be studied separately according to the experimental results. Thus, the contribution analysis-like methods can be a useful tool to calibrate mathematical models by quantifying nonlinear properties and comparing them with the experimental findings. The new parameters are incorporated in the model given in the Appendix section.

We applied our method for the ventricular cardiomyocytes but the contributions of detailed molecular identities of the membrane currents change dramatically within the cell type (heart, neuron, pancreatic beta cells, …), within the species (human, rabbit, rat, …), location within the heart (atrial, ventricular, …), within the developmental stage (newborn, adults, …), and in response to hormones and drugs (ISO (isoproterenol), SO_2_ (sulfur dioxide)) [40]. For example, the role of rapid delayed rectifier was negligible in adult rat ventricular cells, but IKr is one of the major currents in human AP cells [41]. Similarly, we expect larger contribution of IKs in guinea pigs than the human and dog cells. We can quantify the regional contributions and strengths and the differences in the features of the channels, with the method defined here. The developed method here should be a basis for the studies that have been done in this area. For example, it can be used to analyze other types of AP models such as pancreatic beta cell or atrial heart cell, or we can compare the healthy ventricular cell with hypertrophic ventricular cell in terms of the channel contributions. On the other hand, comparing the contribution values of the ion channels with the biophysical findings that have been described in the literature could be a new proof for the consistency of the mathematical models.

The next thing that can be done is to verify this method for each gating variable in dynamic clamp set-up. Dynamic clamp enables us to real-time interface between cell model and real cell to observe the role of each ionic current [42]. It is possible to see the contribution of each gating variable to the real cell AP instead of model AP with this experimental protocol. The newly defined quantitative analysis method here gives a possibility to perform these types of experiments because we measure the contribution values directly from a membrane voltage, different from the other quantitative methods to analyze membrane voltage defined before. However, these works are beyond the scope of this paper.

The contribution analysis method can measure the contribution of each dynamic to every region of AP. However, there is a trade-off between the correctness of the contribution values and the speed of the computer program. The smaller determination of time steps for solving differential equations the more correct contribution values we can get. For this reason, the time step was chosen as small as possible (*dt* = 0.001 ms) in this work. This leads to approximately 12 minutes running time of the computer program for a computer with 2.80 GHz processor. This time step is enough to see even the contribution of small T-Type Ca^2+^ current and rapidly activated K^+^ current, but it is not enough to see which Na^+^ gating variable contributes to the occurrence of very small late current in the plateau phase. For this reason, all contributions were also observed with *dt* =0.0001 ms and the dynamics that contribute to the occurrence of late Na^+^ current were also determined, the other contribution values didn’t change too much. This shows *dt* =0.0001 ms should be enough to see the correct contribution value and the method is stable enough. Another limitation is that the contribution analysis method is not able to measure the contributions of time-independent currents like pump currents. For these, other quantification analysis from the literature like dominant scale analysis can be applied.

## Supplementary Material

Supplemental Material

## Code availability:

Code is available upon request.

## APPENDIX

**Model Summary**

**Membrane currents**

**Fast** $Na^{+}$ **current**, $I_{Na}$

$$I_{Na}=\bar{g_{Na}}n^{3}n_{f}n_{s}\left(V-E_{Na}\right)$$

**L-type** $Ca^{2+}$ **current**, $I_{Ca\_L}$

$$I_{Ca\_L}=\bar{g_{CaL}}l\left[l_{Ca_{i}}l_{f}+\left(1-l_{Ca_{i}}\right)l_{s}\right]\left(V-E_{Ca}\right)$$

$$l_{\infty }\left(V\right)=\frac{1}{1+e^{-0.18V-2.783}},l_{f\infty }\left(V\right)=\frac{1}{1+e^{0.2V+4.9789}},l_{s\infty }\left(V\right)=\frac{1}{1+e^{0.2V+6.5466}}$$

$$\tau_{l}\left(V\right)=\frac{1}{0.321e^{-0.0253V+6.696}+10.83e^{-0.0172V+1.297}}$$

For V≤−24

$$\tau_{l_{f}}\left(V\right)=\frac{1}{0.000277e^{-0.0952V-1.5047}+0.954e^{0.110V-0.595}}$$

$$\tau_{l_{s}}\left(V\right)=\frac{1}{13204.6e^{-556.709V-51.1203}-8959.66e^{6523.77V+950.013}}$$

For V>−24

$$\tau_{l_{f}}\left(V\right)=\frac{1}{-0.1714e^{0.0212V+2.3225}+2.2994e^{0.0207V-0.2455}}$$

$$\tau_{l_{s}}\left(V\right)=\frac{1}{0.0021e^{0.0866V+5.0704}+413.37e^{1.5380V+19.050}}$$

$${l_{Ca_{i}}}_{\infty }=\frac{1}{1+20{\left[Ca\right]}_{ss}}$$

**T-type** $Ca^{2+}$ **current**, $I_{Ca\_T}$

$$I_{Ca\_T}=g_{CaT}c_{t}c_{ti}\left(V-E_{Ca}\right)$$

**Transient** $K^{+}$ **current**, $I_{t0}$

$$I_{t0}=\bar{g_{t0}}k_{t}(ak_{tf}+bk_{ts})\left(V-E_{K}\right)$$

$$k_{t_{\infty }}\left(V\right)=\frac{1}{1+e^{-0.0875V-0.9281}},k_{t_{f_{\infty }}}\left(V\right)=\frac{1}{1+e^{0.1452V+6.5804}},k_{t_{s_{\infty }}}\left(V\right)=\frac{1}{1+e^{0.1452\;V+6.5804}}$$

$$\tau_{k_{t}}\left(V\right)=\frac{1}{\;0.209e^{-0.1\;V-4.629}+0.0025e^{0.035V+4.592}}$$

ForV≤−40

$$\tau_{k_{t_{f}}\;}\left(V\right)=\frac{1}{\;0.392e^{0.1038\;V+1.516\;}+9.4\times 10^{7}e^{-0.1V+0.12}}$$

For V>−40

$$\tau_{k_{t_{f}}\;}\left(V\right)=\frac{1}{\;-0.594e^{0.00089\;V-0.014}+0.1809e^{0.00084V+1.2225}}$$

$$\tau_{k_{t_{s}}}\left(V\right)=\frac{1}{\begin{array}{c} 0.9434\;e^{0.0935V+0.8956}+0.0113e^{-0.02V-5.5541}\\ +414\times 10^{5}e^{-0.216V-46.4}-3.2\times 10^{-12}e^{0.093V+27.373} \end{array}}$$

**Inward rectifier** $K^{+}$ **current**, $I_{K1}$

$$I_{K1}=g_{K1}k_{1\infty }\left(V-E_{K}\right)$$

$$k_{1_{\infty }}\left(V\right)=\frac{1}{1+e^{0.081V+7.0423}}$$

**Rapidly activated outward rectifier** $K^{+}$ **current**, $I_{Kr}$

$$I_{Kr}=g_{Kr}rr_{i}\left(V-E_{K}\right)$$

$$r_{\infty }\left(V\right)=\frac{1}{1+e^{-0.55417V+23.7002}}$$

$$\tau_{r}\left(V\right)=\frac{1}{0.0036\;e^{0.15514V+1.2799\;}+0.0022\;e^{0.018508\;V+0.13531\;}}$$

$$\tau_{r_{i}}\left(V\right)=32.774$$

**Slowly activated outward rectifier** $K^{+}$ **current**, $I_{Ks}$

$$I_{Ks}=\bar{g_{Ks}}s^{2}\left(V-E_{K}\right)$$

$$s_{\infty }\left(V\right)=\frac{1}{1+e^{-0.067V-1.1484}}$$

$$\tau_{s}\left(V\right)=\frac{1}{0.0111\;e^{0.006V+1.239\;}+0.00741\;e^{-0.043\;V+0.3405\;}}$$

**Hyperpolarizing-activated current**, $I_{f}$

$$I_{f}=g_{f}y\left[f_{Na}\left(V-E_{Na}\right)+f_{K}\left(V-E_{K}\right)\right]$$

$$y_{\infty }\left(V\right)=\frac{1}{1+e^{0.095V+13.225}}$$

$$\tau_{y}\left(V\right)=\frac{1}{1.118*10^{-4}\;e^{0.0352V+2.819\;}+5.624*10^{-4}\;e^{-0.070\;V-5.638\;}}$$

**Background currents**

$I_{Xb}=g_{xb}\left(V-E_{X}\right),X=Na^{+},Ca^{2+},K^{+},Cl^{-}$

**Sarcolemmal** $Ca^{2+}$ **pump current**, $I_{pCa}$

$$I_{pCa}=g_{pCa}\frac{{\left[Ca^{2+}\right]}_{i}}{0.0005+{\left[Ca^{2+}\right]}_{i}}$$

$Na^{+}$-$Ca^{2+}$ **exchanger (NCX) current**, $I_{NaCa}$

**Na/K Pump current**, $I_{NaK}$

$Ca^{2+}$ **handling mechanism**

*Intracellular Ca^2+^ diffusion and buffering*

**Ryanodine receptor RyR channel**

$$\frac{dP_{O_{1}}}{dt}=k_{a}^{+}[Ca_{i}^{2+}{]}_{i}^{n}P_{C_{1}}-k_{a}^{-}P_{O_{1}}-k_{b}^{+}[Ca_{i}^{2+}{]}_{i}^{m}P_{O_{1}}+k_{b}^{-}P_{O_{2}}-k_{c}^{+}P_{O_{1}}+k_{c}^{-}P_{C_{2}}$$

$$\frac{dP_{O_{2}}}{dt}=k_{b}^{+}[Ca_{i}^{2+}{]}_{i}^{m}P_{O_{1}}-k_{b}^{-}P_{O_{2}}$$

$$\frac{dP_{C_{1}}}{dt}=-k_{a}^{+}[Ca_{i}^{2+}{]}_{i}^{n}P_{C_{1}}+k_{a}^{-}P_{O_{1}}$$

$$\frac{dP_{C_{2}}}{dt}=k_{c}^{+}P_{O_{1}}-k_{c}^{-}P_{C_{2}}$$

**Intracellular ion fluxes and concentrations**

$$J_{release}=v_{rel}\left(P_{o1}+P_{o2}\right)\left({\left[Ca^{2+}\right]}_{JSR}-{\left[Ca^{2+}\right]}_{ss}\right)$$

$$J_{xfer}=\frac{{\left[Ca^{2+}\right]}_{ss}-{\left[Ca^{2+}\right]}_{i}}{\tau_{xfer}}$$

$$J_{TR}=\frac{{\left[Ca^{2+}\right]}_{NSR}-{\left[Ca^{2+}\right]}_{JSR}}{\tau_{tr}}$$

$$J_{uptake}=\frac{v_{maxf}{\left({\left[Ca^{2+}\right]}_{i}/K_{fb}\right)}^{N_{fb}}-v_{maxr}{\left({\left[Ca^{2+}\right]}_{NSR}/K_{rb}\right)}^{N_{rb}}}{1+{\left({\left[Ca^{2+}\right]}_{i}/K_{fb}\right)}^{N_{fb}}+{\left({\left[Ca^{2+}\right]}_{NSR}/K_{rb}\right)}^{N_{rb}}}$$

$$J_{leak}=v_{leak}\left({\left[Ca^{2+}\right]}_{NSR}-{\left[Ca^{2+}\right]}_{i}\right)$$

$$\frac{d{\left[Ca^{2+}\right]}_{JSR}}{dt}=\beta_{JSR}\left\{J_{TR}-J_{release}\right\}$$

$$\frac{d{\left[Ca^{2+}\right]}_{NSR}}{dt}=\left(J_{up}-J_{leak}\right)\frac{V_{myo}}{V_{NSR}}-J_{tr}\frac{V_{JSR}}{V_{NSR}}$$

$$\frac{d{\left[Ca^{2+}\right]}_{ss}}{dt}=\beta_{ss}\left\{J_{rel}\frac{V_{JSR}}{V_{ss}}-J_{xfer}\frac{V_{myo}}{V_{ss}}-I_{CaL}\frac{A_{cap}C_{m}}{2V_{ss}F}\right\}$$

$$\frac{d{\left[Ca^{2+}\right]}_{i}}{dt}=\beta_{i}\{J_{leak}+J_{xfer}-J_{up}-J_{trpn}-(I_{BCa}-2I_{NaCa}+I_{Cap})\frac{A_{cap}C_{m}}{2V_{myo}F}\}$$

$$\beta_{i}={\left\{1+\frac{{\left[CMDN\right]}_{tot}K_{m}^{CMDN}}{{\left(K_{m}^{CMDN}+{\left[Ca^{2+}\right]}_{i}\right)}^{2}}\right\}}^{-1}$$

$$\beta_{ss}={\left\{1+\frac{{\left[CMDN\right]}_{tot}K_{m}^{CMDN}}{{\left(K_{m}^{CMDN}+{\left[Ca^{2+}\right]}_{ss}\right)}^{2}}\right\}}^{-1}$$

$$\beta_{JSR}={\left\{1+\frac{{\left[CSQN\right]}_{tot}K_{m}^{CSQN}}{{\left(K_{m}^{CSQN}+{\left[Ca^{2+}\right]}_{JSR}\right)}^{2}}\right\}}^{-1}$$

$$J_{trpn}=k_{htrpn}^{+}{\left[Ca^{2+}\right]}_{i}\left({\left[HTRPN\right]}_{tot}-\left[HTRPNCa\right]\right)-k_{htrpn}^{-}\left[HTRPNCa\right]+k_{ltrpn}^{+}{\left[Ca^{2+}\right]}_{i}\left({\left[LTRPN\right]}_{tot}-\left[LTRPNCa\right]\right)-k_{ltrpn}^{-}\left[LTRPNCa\right]$$
