# Supplementary material for: Quantitative roles of ion channel dynamics on ventricular action potential
Source: Channels (Austin). 2021 Jul 16;15(1):465–82. doi: 10.1080/19336950.2021.1940628 (PMC8288042; doi:10.1080/19336950.2021.1940628)
Supplement: Supplemental Material [file KCHL_A_1940628_SM5162.zip › supp.pdf]

Supplementary Figures

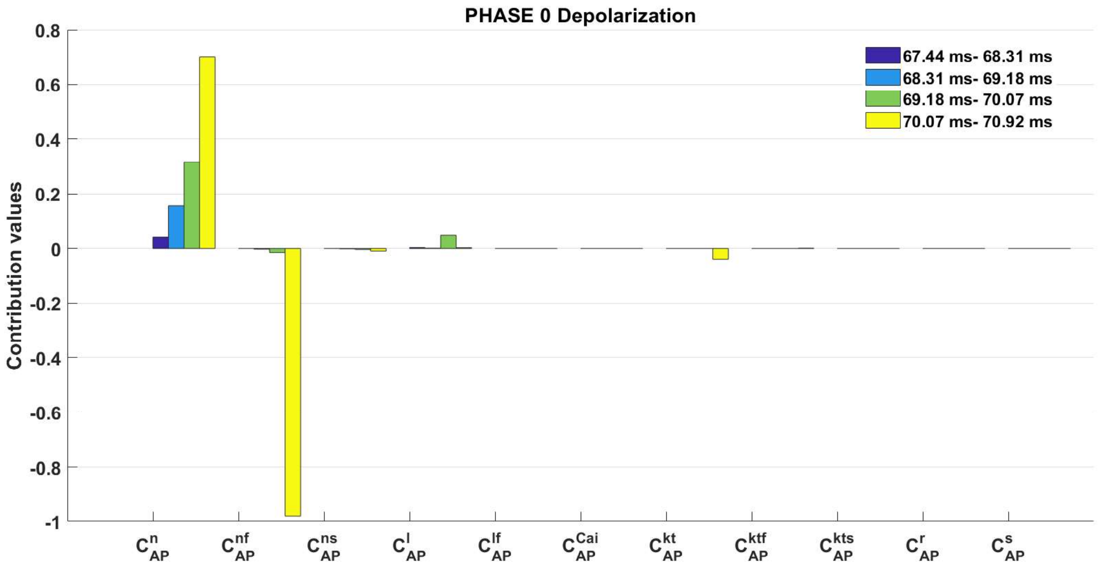

SF1 : Contribution quantification during ventricular AP depolarization phase 0 is divided into four IoI regions as color coded and the related time intervals are shown with I1,I2,I3 and I4. From left, the dynamics of the currents contributing the depolarization phase are: activation of Na^{+} current C_{AP}^{n}, fast-inactivation of Na^{+} current C_{AP}^{nf}, slow inactivation of Na^{+} current C_{AP}^{ns}, activation of L-type Ca^{2+} current C_{AP}^{l}, fast inactivation of L-type Ca^{2+} current C_{AP}^{lf}, Ca^{2+}-dependent inactivation of L-type Ca^{2+} current C_{AP}^{Cai}, activation of transient K^{+} current C_{AP}^{kt}, fast-inactivation of transient K^{+} current C_{AP}^{ktf} ,fast-inactivation

of transient K^+ current C_{AP}^{kts}, rapidly-activated outward rectifier K^+ current activation C_{AP}^{r} and slowly-activated outward rectifier K^+ current activation C_{AP}^{s}.

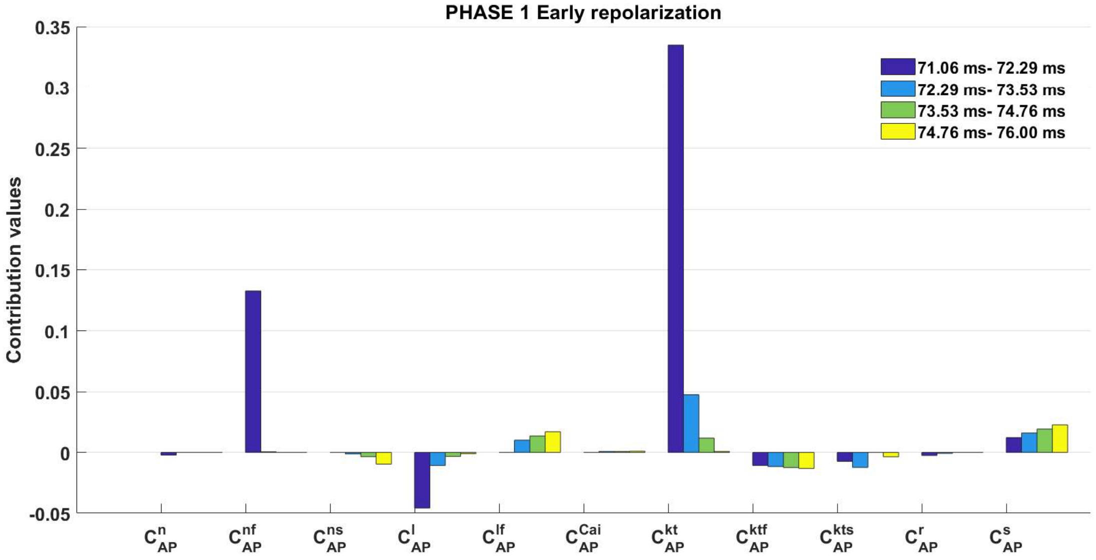

SF2: Contribution quantification during ventricular AP early repolarization. Phase 1 is divided into four IoI regions as color coded and the related time intervals are shown with I1,I2,I3 and I4. From left, the dynamics of the currents contributing the depolarization phase are: activation of Na^+ current C_{AP}^{n}, fast-inactivation of Na^+ current C_{AP}^{nf}, slow inactivation of Na^+ current C_{AP}^{ns}, activation of L-type Ca^{2+} current C_{AP}^{l}, fast inactivation of L-type Ca^{2+} current C_{AP}^{lf}, Ca^{2+}-dependent inactivation of L-type Ca^{2+} current C_{AP}^{Cai}, activation of transient K^+ current C_{AP}^{kt}, fast-inactivation of transient K^+ current C_{AP}^{ktf},fast-inactivation of transient K^+ current C_{AP}^{kts}, rapidly-activated outward rectifier K^+ current activation C_{AP}^{r} and slowly-activated outward rectifier K^+ current activation C_{AP}^{s}.

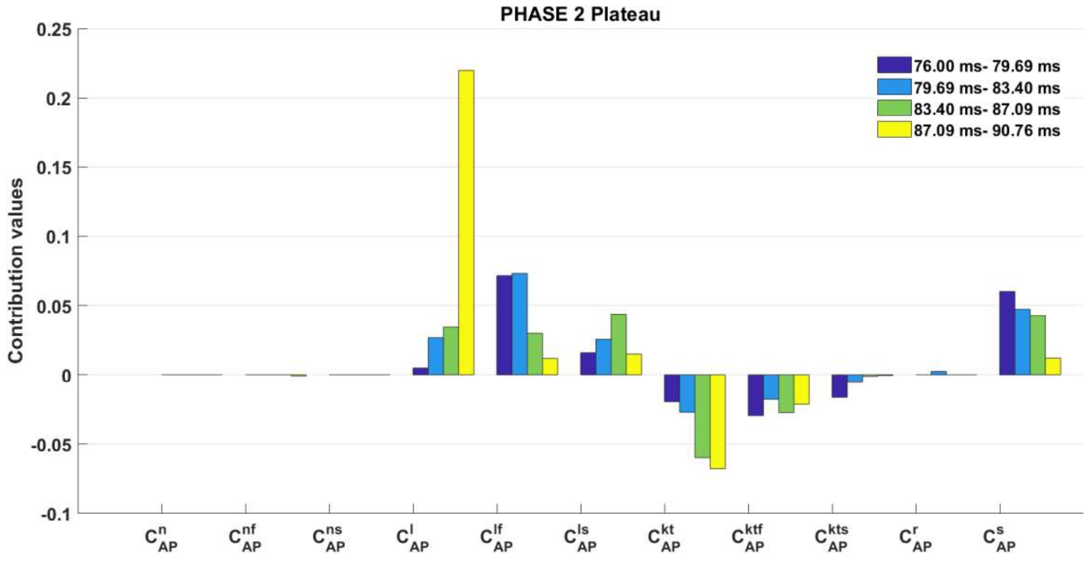

SF3: Contribution quantification during ventricular AP plateau..Phase 2 is divided into 4 IoI regions as color coded and the related time intervals are shown with I1,I2,I3 and I4. From left, the dynamics of the currents contributing the depolarization phase are: activation of Na^+ current C_{AP}^{n}, fast-inactivation of Na^+ current C_{AP}^{nf}, slow inactivation of Na^+ current C_{AP}^{ns}, activation of L-type Ca^{2+} current C_{AP}^{l}, fast inactivation of L-type Ca^{2+} current C_{AP}^{lf}, Ca^{2+}-dependent inactivation of L-type Ca^{2+} current C_{AP}^{Cai}, activation of transient K^+ current C_{AP}^{kt}, fast-inactivation of transient K^+ current C_{AP}^{ktf},fast-inactivation of transient K^+ current C_{AP}^{kts}, rapidly-activated outward rectifier K^+ current activation C_{AP}^{r} and slowly-activated outward rectifier K^+ current activation C_{AP}^{s}.
